# Pemphigus vulgaris after the second dose of COVID-19 vaccination: a case report

**DOI:** 10.1186/s13256-023-04055-0

**Published:** 2023-07-27

**Authors:** Naram Khalayli, Abdullah Omar, Mayssoun Kudsi

**Affiliations:** 1 Faculty of Medicine, Damascus University, Damascus, Syria; 2 Faculty of Medicine, Syrian Private University, Damascus, Syria; 3 Department of Dermatology and Venerology, Damascus hospital, Damascus, Syria; 4 Rheumatology Department, Damascus University, Damascus, Syria

**Keywords:** COVID-19, Pemphigus vulgaris, Vaccination, Auto immune bullous diseases

## Abstract

**Background:**

As many people worldwide have been vaccinated, more triggered autoimmune bullous diseases have been noticed. We reported a case of new-onset pemphigus Vulgaris after COVID-19 vaccinations.

**Case presentation:**

A 50 years old Syrian female presented with multiple erosions on her extremities, in addition to oral erosions and genital ulcers, after the 2nd dose of the mRNA COVID-19 vaccine. The lesions were multiple tenders and well-defined, reddish erythematous oral ulcers. At the same time the Skin examination showed hyper-pigmented patches over the extremities, a sequel of ruptured bullae. Pemphigus Vulgaris was diagnosed by histology. She continued on 35 mg/day of prednisone for another 2 weeks.

**Conclusions:**

Pemphigus is not a contraindication to vaccination, although it may be worse or present as a first onset.

## Introduction

Pemphigus Vulgaris is a rare, fatal, autoimmune disease. It occurred due to the disruption of intercellular keratinocytes junctions by anti-desmoglein antibodies [1].

It is usually affecting middle-aged patients. It causes mucous membranes and cutaneous blisters. It may be triggered or aggravated by some treatment or vaccines like hepatitis B, influenza, rabies, and tetanus vaccination [1].

About half of the patients have only oral erosions, which often precede skin involvement. Dysphagia is common due to esophagus involvement. In addition, the Cutaneous bullae leave a raw area with crusting. The Erosions often become infected. Fluid and electrolyte loss may happen when large body areas are affected. This disorder must be differentiated from other conditions that cause chronic oral ulcers and skin bullous [2].

The diagnosis is confirmed by lesional and surrounding normal skin biopsy that shows IgG autoantibodies binding the keratinocyte’s cell surface by immunofluorescence. Desmoglein 1 and desmoglein three transmembrane glycoproteins autoantibodies can be found [2].

Vaccines are still the most effective and protective method against COVID-19 infection. However, there are cases of new onset or flare of pemphigus following COVID-19 vaccination [3].

We are reporting A triggered bullous lesion after the second vaccine dose.

## Case presentation

A 50 years old Syrian female presented with multiple erosions on her extremities, oral erosions, and genital ulcers 10 days after the second dose of the mRNA COVID-19 vaccine. No previous medical history or diseases, nor family history. She had received 35 mg/day of prednisone 1 week before her presentation to our department.

**Fig. 1:**
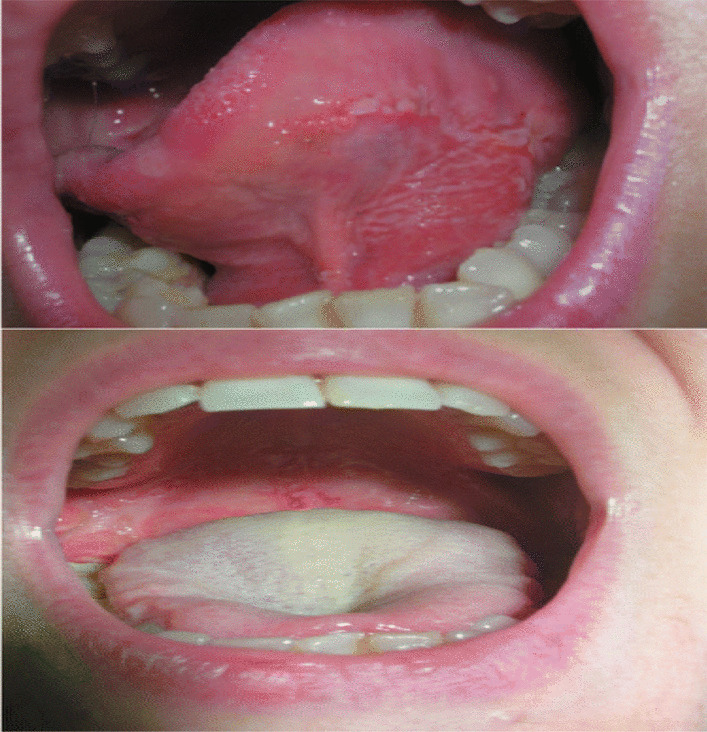
Oral involvement

Intraoral examination revealed multiple tenders and well-defined reddish erythematous ulcers (Fig. 1).

**Fig. 2:**
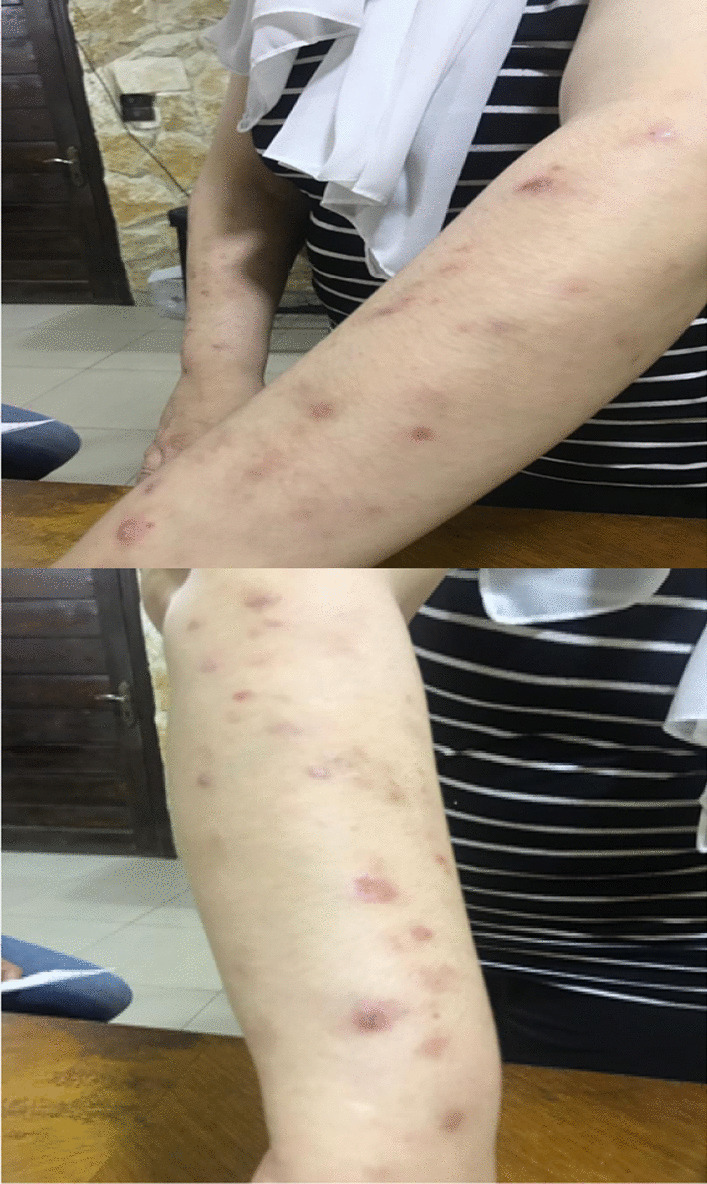
Skin involvement after initiation of steroids, at the beginning

Skin examination showed hyper-pigmented circular patches over the extremities, especially on the upper arms, as well as a sequel of ruptured bullae (Fig. 2).

**Fig. 3:**
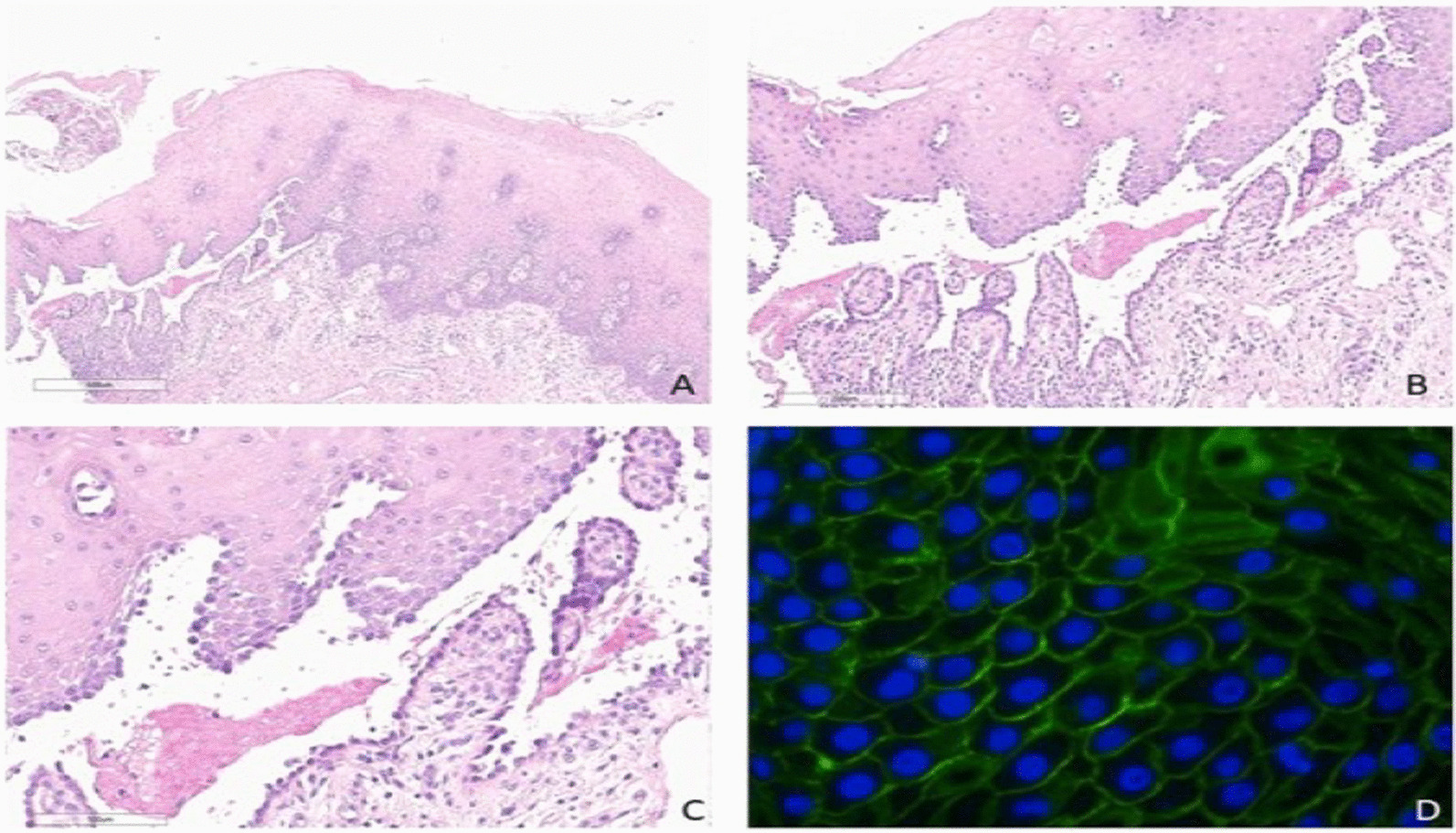
the histologic findings, and the immunohistochemistry

A skin biopsy had been taken from the fresh vesicles. The histologic findings were a row of “tomb-stone” appearance and suprabasal cleft formation of basal cells. Also, Direct immunofluorescence showed deposition of IgG in the epidermis (Fig. 3).

According to the clinical and histopathological findings, pemphigus Vulgaris was diagnosed.

**Fig. 4:**
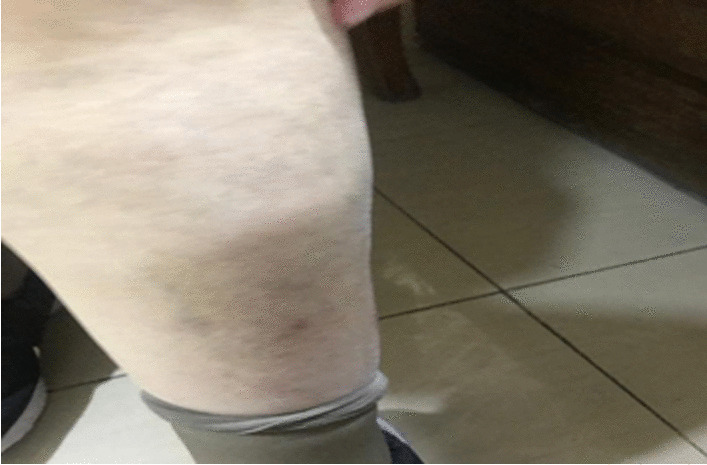
The responded skin manifestation

We prescribed a topical steroid for the oral ulcers, and she continued with 35 mg/day of prednisone for another 2 weeks. A good response was achieved in oral and skin manifestations (Fig. 4).

## Discussion

COVID-19 vaccination may trigger an immunological response. The hyper-stimulated state of the immune system triggers the onset of autoimmune dysregulation in genetically predisposed individuals. On the other hand, vaccine-induced immunity has both a cell-mediated and humoral response.

No research has confirmed the mechanisms for vaccine-induced autoimmune disorders.

The robust T and B immunocytes, and the cytokine cascades, provide anti-COVID-19 protective efficacy in individuals. Pemphigus is a TH2 cell-driven disease, a complex TH17/TFH17 cell–dominated disease, and these cells produce lineage-specific cytokines like IL-4, IL-5, IL-17, and IL-21, and desmoglein-specific antibodies [1].

Certainly, the exact etiology and mechanisms behind pemphigus and vaccine are still under investigation [2].

When reviewing the previous data concerning our case, we found that most cases were diagnosed as Bullous Pemphigoid, Linear IgA Bullous Dermatosis, and Pemphigus Vulgaris, and finally as Pemphigus Foliaceus.-, in older ages and with the predominance of males. All patients were treated with topical and systemic corticosteroids, with or without immunosuppressive drugs, with a good clinical response in every case.

The disease may develop within 3 days to 3 weeks following all types of COVID-19 vaccination; after the first or second dose injection, the vaccines may aggravate the pemphigus course (Table 1), and all of these are compatible with our case.

**Table 1:** Published data of pemphigus onset/flare following COV19 vaccination

Study	Age	Sex	Technology/Platform	Dose of vaccine	Diagnosis	New onset or flare	Latency
Solimani *et al. *[3]	40	F	mRNA	1	PV	New onset	5 days
Thongprasom *et al.* [4]	38	F	Modified chimpanzee adenovirus	1	Oral pemphigus	New onset	1 week
Koutlas *et al.* [5]	60	M	mRNA	2	PV	New onset	7 days
Singh *et al.* [6]	44	M	Modified chimpanzee adenovirus	2	PV	New onset	1 week
Akoglu *et al.* [7]	69	F	Inactivated	2	PV	New onset	1 week
Hali *et al.* [8]	50	F	mRNA	1	PF	New onset	15 days
	58	F	mRNA	1	PV	New onset	1 month
Lua *et al.* [9]	83	M	mRNA	2	PF	New onset	2 days
Calabria *et al.* [10]	60	F	mRNA	2	PV	New onset	7 days
Knechtl *et al.* [1]	89	M	mRNA	2	PV	New onset	1 month

Clinical trials have shown that COVID-19 vaccines are Efficacious in preventing symptomatic COVID-19 infection and are well tolerated with acceptable side effects. In contrast, pemphigus is rare in the general population [6].

## Conclusion

Pemphigus is not a contraindication to vaccination, although it may be worse or present as a first onset.

## Data Availability

Not applicable.
